# Heat and Drought Co-Stress During Microgametogenesis Altered Pollen Development, Fertility and Grain Production in Two-Rowed Winter Barley

**DOI:** 10.3390/plants15152360

**Published:** 2026-07-31

**Authors:** Katalin Jäger, Emmanuel Asante Jampoh, Dorina Babinyec-Czifra, Zoltán Kristóf, Beáta Barnabás

**Affiliations:** 1 Agricultural Institute, HUN-REN Centre for Agricultural Research, 2462 Martonvásár, Hungary; emmanuel.jampoh@atk.hun-ren.hu (E.A.J.); babinyec.dorina@gmail.com (D.B.-C.); barnabas.beata@gmail.com (B.B.); 2 Doctoral School of Horticultural Sciences, MATE Hungarian University of Agriculture and Life Sciences, 2100 Gödöllő, Hungary; 3 Doctoral School of Biology, Institute of Biology, ELTE Eötvös Loránd University, 1117 Budapest, Hungary; 4 Plant Anatomy Department, Institute of Biology, ELTE Eötvös Loránd University, 1117 Budapest, Hungary; drkristofz@gmail.com

**Keywords:** barley, heat and drought co-stress, microgametogenesis, hydrogen peroxide, antioxidant enzyme activity, pollen ultrastructure, pollen viability, fertility, grain production

## Abstract

The effects of concurrent heat and drought on phenology, antioxidant enzyme activity, anther and pistil development, anatomy, pollen function and grain production were studied to characterize stress responses in two barley varieties with contrasting tolerance. Plants were grown under optimal environmental conditions until the mid-uninucleate stage of microspore development, then subjected to water withholding and temperatures 10 °C above the optimum during microgametogenesis. HD co-stress shortened the duration of pollen development and tapetum degradation by 3 days. In Lambada leaves, increased GST activity, together with maintained CAT, APX, and GR activities and flavonoid content, was associated with higher pollen viability, fertility, and grain production under HD co-stress, suggesting that enhanced antioxidant capacity may contribute to improved stress tolerance. Compared with Spinner, HD-stressed Lambada pollen maintained higher starch accumulation and better-preserved ultrastructure, whereas Spinner pollen contained smaller starch granules and exhibited swollen mitochondria with ruptured cristae, curved endoplasmic reticulum with dilated cisternae, and numerous autophagosomes and autophagic vacuoles throughout the cytoplasm. Spatiotemporal variation in pollen viability, fertility, and grain production was associated with both the developmental stage of microspores and pollen cells and the position of florets along spikes under HD co-stress.

## 1. Introduction

Barley (*Hordeum vulgare* L.), a member of the Poaceae family, covered 44 Mha, had an average yield of 3.1 t/ha, and produced 141 million tons in 2024 [1]. It is the fourth most-produced cereal in the world, after corn, wheat, and rice. Grown under various agro-climatic conditions, barley is the cereal crop with the widest and most diverse geographical distribution [2] and is used as food, fodder and malting grain [3]. Nonetheless, the impacts of climate change pose a risk to barley production due to expected reductions in precipitation and an increase in average air temperature.

Plants are frequently exposed to multiple abiotic stressors simultaneously [4,5], each with the potential to exacerbate the impact of the others and negatively affect plant growth and yield [6,7]. Among these adverse abiotic factors, increased temperature and water deficit, often co-occurring worldwide, are the major and most destructive stress factors impacting crop growth, development, and yield [8,9,10,11]. Projections indicate that by the end of the 21st century, global surface temperatures are expected to rise by 1.5 °C to 4.4 °C above pre-industrial levels, depending on greenhouse gas emission scenarios [12,13]. The risk of yield loss from drought is expected to rise, with potential impacts of 18.1–19.4% for rice, 9–12% for wheat, 5.6–6.3% for maize, and 15.1–16.1% for soybean by the end of the 21st century [14]. Nonetheless, the co-occurrence of high temperature and water deficit is expected to increase in both frequency and severity under the ongoing climate change regime [15].

The ability of a plant to tolerate and recover from multiple stresses influences its growth, development, and yield [10]. Consequently, as sessile organisms, plants have evolved sophisticated defense mechanisms that enable them to withstand adverse conditions and to ensure survival and reproduction [8,16]. The plant response to combined abiotic stresses is unique and cannot be inferred from responses to a single abiotic stress factor [10,17,18]. Thus, a combination of heat and drought stress has a more harmful effect on crop growth and productivity than either applied alone [19,20,21], as evident in tobacco [17] and maize [7]. This highlights the importance of studying stress combinations in plants to enhance crop tolerance [11].

Agriculture, to a large extent, relies on the success of plant reproduction, which is strongly influenced by environmental conditions [22]. Hence, male and female gamete development, fertilization, and grain development should proceed without perturbation [8]. Reproductive development is sensitive to both high temperature and water deficit, although each stressor affects reproductive organs and cells in distinct ways [7,23]. Drought triggered abnormal anther development, ovule abortion, and failure of flowers to open, as well as reduced pollen viability and filament elongation in wheat and *Arabidopsis thaliana* [24,25]. Moreover, supraoptimal temperature reduced fertility and seed set in spring barley [26] and inhibited the biochemical processes of seed filling in lentils [27]. Increased temperature limited pollen viability in wheat, reduced stigma receptivity in maize, and reduced pollen attachment to the stigma in *Arabidopsis thaliana* [10,28,29,30]. Furthermore, heat stress reduced spikelet fertility and grain set in wheat [31]. During reproductive development, plants are more affected by HD-induced co-stress than during vegetative phenophases [19]. Compared with the large number of studies on the effects of individual heat and drought on reproductive development, few studies examine their combined effects on reproductive processes. Reduced pollen functionality in wheat, chickpeas and rice [28,32,33,34] (for a review, see [8,35]), structural alterations in reproductive organs in wheat [28], and weak sink strength of developing caryopses in wheat and rice [36,37] are known leading causes of HD-induced production loss.

Heat and drought co-stress usually leads to excessive accumulation of reactive oxygen species (ROS), which function as signaling molecules and are toxic by-products of aerobic metabolism, causing severe cellular damage [28,38]. Through evolution, plants have evolved both enzymatic and non-enzymatic antioxidant defense strategies [39] to protect cellular components from oxidative stress damage [40]. Lower ROS accumulation, paired with increased antioxidant capacity in plants, was linked to improved tolerance to combinations of abiotic stresses in Arabidopsis [5,6]. Excessive ROS production has been linked to impaired reproductive organ function and to the development of gametophytes [28,41]; therefore, maintaining a balance between ROS production and scavenging, is mediated by antioxidant enzymes and molecules, is crucial for the development of both male and female gametophytes [42]. Additionally, to mitigate oxidative damage caused by elevated ROS levels, plants may activate autophagy, a conserved cellular degradation pathway responsible for removing damaged or toxic cellular components [43]. Impairment of autophagy can adversely affect plant physiology and compromise stress tolerance [44]. Likewise, autophagy has been implicated in male gametophyte development and fertilization in plants, which involves the timely elimination of damaged or dysfunctional organelles and proteins [45,46]. Hence, autophagy is essential for proper tapetal degradation during pollen maturation and for the maintenance of pollen development under abiotic stress conditions [47].

The aim of this study was to investigate the physiological, biochemical, and developmental mechanisms underlying barley tolerance to combined heat and drought stress during microgametogenesis by comparing barley varieties with contrasting tolerance. We hypothesized that a winter barley genotype with greater tolerance to combined heat and drought stress maintains higher leaf water status and a more effective antioxidant defense, resulting in lower oxidative damage than the sensitive variety. We further hypothesized that these physiological and biochemical advantages preserve reproductive development by reducing structural damage to anthers and pollen cells, ultimately supporting reproductive function under combined stress. In contrast, the sensitive genotype was expected to exhibit greater water deficit, increased hydrogen peroxide accumulation, weaker antioxidant responses, and more severe reproductive impairment, linking reduced stress tolerance with disrupted microgametogenesis, pollen function, and loss of fertility and grain production.

## 2. Results

### 2.1. HD Co-Stress During Microgametogenesis Triggered Phenological Alterations and Affected Tillering

Under optimal (control) conditions, the period from the MU stage of microspore development to flowering lasted 8 days in the main shoots of both Lambada and Spinner plants. HD co-stress reduced this interval by 3 days in both varieties. There was no significant difference in the number of culms at the MU stage of microspore development between the varieties, with 3.1 ± 0.7 for Lambada and 3.0 ± 0.7 for Spinner. During microgametogenesis, the number of culms increased in the controls of both varieties and in HD co-stressed Lambada plants. However, compared with their respective controls, HD co-stressed Spinner plants produced 14% fewer culms between the MU stage and flowering (Table 1). While control plants of both varieties had fully emerged spikes at anthesis, the treatment hindered spike protrusion from the leaf sheaths. Specifically, the number of culms with partially emerged spikes decreased by 33% in Lambada and by 65% in Spinner. In treated Spinner plants, only a few main spikes emerged above the auricles of the flag leaf, whereas the tiller spikes remained completely enclosed within the leaf sheaths. The number of culms (fertile culms) at ripening ranged from 11–18 (5–11) for Lambada control, 11–16 (5–13) for Lambada-treated, 10–14 (4–10) for Spinner control, and 10–16 (8–13) for Spinner HD co-stressed plants. Compared with Spinner, the mean number of Lambada culms and productive culms at ripening was significantly higher under control conditions. Although HD co-stress did not induce additional tillering in either genotype, the proportion of productive Spinner culms increased by 60% (Table 1).

### 2.2. Stress-Induced Decline in the Volumetric Water Content of the Soil and Relative Water Content of the Leaves

The volumetric water content (VWC) of the soil in the Lambada and Spinner pots was 35.9 ± 1.1% and 36.8 ± 0.2%, respectively, corresponding to soil water potentials of −15.4 ± 1.8 kPa and −13.9 ± 0.3 kPa. By the end of the HD co-stress, VWC and soil water potential decreased significantly to 14.5 ± 0.1% (−337.8 ± 7.7 kPa) in Lambada and to 14.4 ± 0.1% (−348.6 ± 8.9 kPa) in Spinner. There was no significant difference in VWC or soil water potential among varieties at either the beginning or the end of HD co-stress. Compared with the flag leaf relative water content (RWC) in the controls (72.1%, 77.5%), simultaneous HD stress caused significant reductions of 16% and 39% in the HD-tolerant Lambada and HD-sensitive Spinner varieties, respectively (Table 2).

### 2.3. Hydrogen Peroxide Accumulated in the Mesophyll Cells of Co-Stressed Plants

A small amount of H_2_O_2_ was detectable by histochemical staining in the leaves of plants grown under optimal conditions (Figure 1A,B). In contrast, HD co-stress induced H_2_O_2_ production in the mesophyll chloroplasts of both varieties (Figure 1C,D). Although H_2_O_2_ content did not differ by genotype in control flag leaves, a notable difference was observed in the co-stress-induced H_2_O_2_ production. Compared with the modest 28% increase in H_2_O_2_ content in Lambada plants, the HD-sensitive Spinner variety showed a significant 7.1-fold increase in this highly reactive compound (Figure 1E). The HD-induced increase in H_2_O_2_ content was strongly negatively correlated with plant RWC (Figure 2).

### 2.4. Severe Decrease in Antioxidant Enzyme Activities

Although there was no genotype-dependent difference in total antioxidant enzyme (AE) activities among barley genotypes under control conditions (Figure 3A), by the 5th day of HD co-stress treatment, the total AE activity of the Spinner variety was 59% lower than that of the Lambada plants. Among the examined AEs, CAT activity in flag leaves was the highest, accounting for 93–95% of total AE activity under both control and HD co-stress conditions. Under HD co-stress, activities of CAT, G-POD, APX, and GR in the flag leaves of the Spinner variety decreased by 59%, 70%, 42%, and 48%, respectively (Figure 3B–E). Meanwhile, the GST activity of the genotype remained unaffected (Figure 3F). In contrast, stress did not influence CAT, APX, and GR activities in Lambada leaves (Figure 3B,D,E); however, it caused a notable 34% decrease in G-POD activity (Figure 3C) and a 52% increase in GST activity (Figure 3F). The activities of total AEs, CAT, G-POD, APX, GR and APX showed strong and very strong positive correlations with RWC at the *p* ≤ 0.01 level (Figure 2). Moreover, the activities of the same antioxidant enzymes and their total activity showed strong negative correlations with H_2_O_2_ content in the flag leaves.

### 2.5. Co-Stress-Induced Changes in Flavonoid Contents

A genotype-dependent difference in total flavonoid content in flag leaves was observed under control conditions, with the Spinner variety having 3.7 times the content of Lambada. The two genotypes responded differently to the treatment: HD co-stress caused a significant 60% decrease in the Spinner genotype, but did not significantly affect Lambada plants (Figure 3G). The flavonoid content of the flag leaves showed a weak negative correlation with H_2_O_2_ accumulation and a negligible correlation with RWC (Figure 2).

### 2.6. Simultaneous Heat and Drought-Induced Changes in the Development and Morphology of Anthers and Pistils

HD co-stress triggered asynchrony in reproductive development and altered the morphology of both male and female sexual organs (Figure 4; Table 3). In control main spikes, anthers turned yellow on the morning of the 8th day after the mid-uninucleate stage of microspore development (DAMU), regardless of floret position on the spike. In the central region of the spikes, anthers protruded from the florets and dehisced at 11 a.m. In contrast, in HD co-stressed main spikes, anthesis began 3 days earlier, so anthers turned yellow on the morning of the 5th day of treatment. In the exposed part of the treated Lambada spikes, anthers protruded from the florets and dehisced at 11 a.m. In contrast, in the treated non-protruded part of Lambada spikes and in almost all Spinner spikes, anthers remained inside the unopened florets.

A genotype-dependent difference in anther length was observed at anthesis under both control and co-stress conditions, with Spinner anthers longer than those of Lambada across all spike regions (Figure 4; Table 3). The extent of the HD-induced reduction in anther size varied by floret position. In HD co-stressed Lambada, the top and basal anthers, as well as those in the basal florets of the Spinner main spikes, were significantly shorter than those in the central florets. Contrary to enhanced anther development, simultaneous HD stress delayed pistil development by 1 day in Spinner florets and by 2 days in Lambada, regardless of spikelet position. Unlike control pistils with feathery stigmas, the HD co-stressed female organs of Lambada were significantly smaller and had shorter stigmas at anthesis across all spike regions. In Spinner, HD co-stress did not significantly affect stigma length, ovary height, or ovary width in the superior or inferior florets. However, a substantial decline in these parameters was observed in the central pistils (Figure 4; Table 3). Stigma length, ovary length and ovary width showed strong positive and significant correlation with RWC (Figure 2).

### 2.7. Position-Specific Effect of Heat and Drought on Starch Accumulation

Acetocarmine staining was used to evaluate the developmental stage of pollen cells at anthesis, providing insight into stress-induced alterations. The presence of spindle-shaped sperm cells confirmed that tricellular pollen isolated from all regions (base, center, top) of both control and HD co-stressed spikes was mature in both varieties at anthesis (Figure 5). Regardless of anther position, starch reserves were abundant in control pollen cells of both genotypes (Figure 5A–C,G–I). HD co-stress applied for 5 days during microgametogenesis reduced starch accumulation, and pollen cells exhibited signs of dehydration (Figure 5D–F,J–L). Genotype- and position-dependent variation in starch accumulation was observed after HD co-stress treatment. Starch deposits in male gametophytes isolated from the base and center of treated Lambada main spikes did not differ from those in the controls (Figure 5D,E). However, pollen from the top florets of the same variety accumulated less starch (Figure 5F). In Spinner, regardless of position, HD co-stressed male gametophytes accumulated significantly fewer starch granules (Figure 5J–L) than their respective controls or treated Lambada pollen cells.

### 2.8. Heat and Drought-Induced Structural Changes in the Anthers and Pollen Cells

A study of the timing of degeneration in anther layers (epidermis, endothecium, middle layer, tapetum) and starch accumulation during microgametogenesis under control and HD co-stress conditions was conducted using light microscopy (Figure 6). As anther development progressed under optimal conditions (Figure 6A–P), degeneration of epidermal cells and endothecial chloroplasts was observed at 7 DAMU in both genotypes (Figure 6G,O). By the 8th DAMU, the cells of the innermost anther layer, the tapetum, were almost completely degenerated, and few Ubisch bodies were visible on the luminal surface of the degenerated tapetum (Figure 6H,P). HD co-stress accelerated degeneration of anther layers, with visible symptoms appearing on the 3rd DAMU in Lambada and on the 4th DAMU in Spinner (Figure 6S,Y).

It accelerated pollen maturation in both varieties by 3 days, thereby reducing the duration and extent of starch accumulation (Figure 6Q,Z). Compared with control anthers, the epidermis and endothecial layers of HD co-stressed anthers were more shrunken, and their protoplasm disappeared by anthesis (Figure 6U,Z). Additionally, the middle layer and tapetum were crushed, leaving only a large number of orbicules visible on the inner surface of the degenerated tapetum. Starch accumulation in control pollen cells was initiated on day 4 in both varieties (Figure 6D,L) and persisted for 4 days; however, a notable genotype-dependent difference in deposition dynamics was observed (Figure 6D–H,L–P). While starch gradually accumulated in the control Spinner male gametophytes, significant starch deposition in Lambada pollen was observed only at 7 DAMU. Compared with the controls, starch granules began accumulating in treated pollen of both varieties a day earlier (Figure 6S,X).

### 2.9. Ultrastructure of Control and Co-Stressed Barley Anthers and Pollen Cells

No genotypic differences were observed in the ultrastructure of control anthers and pollen cells at anthesis. The exothecium contained numerous lipid bodies, and some chloroplasts were still visible in the endothecial cells (Figure 7A). The middle layer disintegrated, and in some areas, remnants of degraded secretory tapetum cells were present. The exine and intine of control pollen had a consistent structure, with both layers remaining uniform around the cells (Figure 7B). A large number of starch granules and Golgi vesicles typical of this developmental stage, along with stacks of rough endoplasmic reticulum and only a few vacuoles, were present in the cytoplasm of vegetative cells (Figure 7B,C). No genotypic difference in the structure of HD co-stressed anthers was observed. A greater number of sporopollenin-containing Ubisch bodies located near the luminal surface was typical of the tapetum (Figure 7D). No undegraded tapetum cells were present in treated anthers. Although the mean exine thickness did not vary with the treatment (d = 0.81 ± 0.08 µm) in either genotype, the intine of treated Lambada and Spinner pollen expanded significantly by 33% and 40%, respectively, compared with the mean intine thickness of the control pollen (d = 0.58 ± 0.1). The intine thickness showed a strong positive correlation with H_2_O_2_ content and a strong negative correlation with RWC, total AE, CAT, G-POD, APX, GR activities, and pollen viability (Figure 2). Regardless of variety, uneven intine thickness was a typical feature of HD-co-stressed pollen cells (Figure 7E,G); however, no significant genotypic difference was observed for this trait. The ultrastructure was heterogeneous; the number of vacuoles increased in the cytoplasm, and myelin-like membrane coiling was typical for treated Lambada pollen (Figure 7F). Many compressed pollen grains were visible in the treated Spinner anthers, which had become polygonal due to dehydration (Figure 7G). Compared with the control, the cytoplasm of treated Spinner pollen contained fewer and smaller starch granules. Swollen mitochondria with ruptured cristae and curved ER with dilated cisternae, which were absent in the control, were observed, along with numerous autophagosomes and autophagic vacuoles scattered throughout the cytoplasm of the sensitive variety (Figure 7G,H). An electron-dense layer formed on the surface of Golgi vesicles in HD co-stressed Spinner pollen (Figure 7H).

### 2.10. Heat and Drought Reduced Pollen Viability

When evaluating the effects of HD stress on pollen viability, plant fertility and grain yield, both the main shoots and tillers should be considered. However, unlike the main shoots, in which the anthers experienced HD co-stress throughout microgametogenesis, the precise timing of flowering and the proportion of viable pollen in HD co-treated tillers could not be determined because, regardless of the variety, the spikes were trapped and anthesis occurred within the leaf sheaths. No genotype-related differences in pollen viability among florets along the main spikes were observed in control plants (Figure 8A).

After concurrent HD stress, Lambada and Spinner pollen viability decreased significantly by 24% and 34%, respectively. Under optimal growth conditions, no location-dependent variation in Lambada pollen viability (mean 81%) was observed (Figure 8B). Control Spinner pollen viability showed significant spatial variation, with viability ratios of 71%, 89%, and 81% in the basal, central, and top florets, respectively. Compared with central anthers, HD co-stress significantly reduced the proportion of viable pollen in both the superior (−31%) and inferior (−29%) florets of Lambada. Spinner pollen experienced a greater stress-induced decrease in viability, with reductions of 50%, 18%, and 39% in the basal, central, and top regions, respectively. Pollen viability showed a strong, significant positive correlation with RWC and with total AE, CAT, G-POD, APX and GR activities (Figure 2). In contrast, pollen viability negatively correlated with H_2_O_2_ content and intine thickness.

### 2.11. Simultaneous Heat and Drought Stress Severely Reduced Fertility and Grain Production in the Main Shoots

Control main shoots showed genotype-dependent variations in fertility ratios, with Spinner plants having a notably lower mean (62.2%) than Lambada (77.9%; Figure 8C). Irrespective of genotype, the main spikes of treated plants had significantly lower fertility than those of their control counterparts. Co-occurring HD stress reduced fertility by 86% and 93% in the main shoots of Lambada and Spinner, respectively. In the control main spikes of both genotypes, fertility was floret-position dependent: central florets had the highest fertility, whereas basal florets had the lowest (Figure 8D). As with the stress-induced, location-dependent loss of pollen viability, the smallest decline in fertility was observed in the central region of the main spikes. None of the Spinner basal florets on the main spikes was fertilized. The varieties did not compensate for the loss of HD-induced fertility by increasing thousand-grain weight (TGW), so grain production in the main shoots decreased by 88% for Lambada and 92% for Spinner (Figure 8E). Under control conditions, the spikelets in the lower third of the main spikes contributed the least to grain production; however, this pattern disappeared under stress conditions (Figure 8F) because HD co-stress induced sterility in the florets at both the base and the top of the Spinner spikes. Fertility of the main spikes was strongly correlated with RWC, AE, CAT, G-POD, APX activities, stigma length and pollen viability (Figure 2). There was a strong negative correlation between the fertility of the main spikes and RWC, as well as between the fertility of the main spikes and intine thickness. Grain production of the main spikes showed a strong positive correlation with RWC, antioxidant enzyme activities, pollen viability and fertility of the main spikes.

### 2.12. HD Co-Stress Altered the Fertility and Grain Production of the Plants

Concomitant heat and drought applied for 5 days reduced the fertility of Lambada and Spinner plants by 24% and 45%, respectively (Figure 9A). For stress-induced reductions in TGW of 23% and 32%, grain production of Lambada and Spinner plants declined by 42% and 52%, respectively (Figure 9B). For individual culms, fertility and grain production depended heavily on the developmental stage at which microspores were exposed to HD co-stress. Early uninucleate microspores and tetrads (1st tillers), diads (2nd and 3rd tillers), and microspore mother cells (MMCs; 3rd and 4th tillers) were isolated from jointing tillers when the microspores in the main shoots of the plants were at the MU stage of development (the start of the 5-day HD co-stress). Although HD co-stress had no effect on tillering, it induced the appearance of 2 and 3 additional spikes with grain set in Lambada (Figure 9C,D) and Spinner (Figure 9E,F), respectively. Nevertheless, the fertility and grain production of those tillers were severely reduced. The 1–6 culms of control Lambada (Figure 9C) and 1–5 culms of control Spinner (Figure 9E) showed the highest fertility ratios, which significantly decreased in the 7th to 11th culms of Lambada and the 6th to 10th culms of Spinner. A similar trend in grain production was observed in Lambada plants (Figure 9D), whereas in the control Spinner, the first two culms produced the most, with subsequent culm production gradually declining (Figure 9F). Taking into account all culms of individual barley plants, there was an HD co-stress-induced genotype-dependent difference in both spikelet fertility and grain production patterns (Figure 9C–F). In HD co-stressed Lambada plants, the main shoots had the lowest fertility ratios and grain production, similar to those of the 9–13 culms (Figure 9C,D). Plant fertility showed a moderate positive correlation with RWC and antioxidant enzyme activities at anthesis (Figure 2). Grain production showed a strong positive correlation with RWC, total AE, CAT, G-POD and APX activities at anthesis. The number of productive culms showed a strong positive correlation with H_2_O_2_ content in the flag leaves at anthesis and a strong negative correlation with antioxidant enzyme activities.

Nevertheless, the fertility of the 4–8 culms and the grain production of the 4–7 culms did not differ significantly from the controls. In the treated Spinner plants, only the fertility of the 6–9 culms reached control values (Figure 9E). A similar pattern was observed in grain production: the yield of the 6th to 8th culms exceeded that of the remaining culms (Figure 9F).

In this study, the terms tolerant and sensitive are used to describe the relative performance of the Lambada and Spinner genotypes under HD co-stress. These designations are based on comparative results in their physiological, biochemical, anatomical, reproductive, and yield responses observed in both our present and previous study [48].

## 3. Discussion

Global warming significantly affects crop phenology by shortening developmental stages, thereby reducing grain production [49]. A combination of water deficit and high temperatures during reproductive development can lead to reproductive failure in plants. In the present study, HD co-stress reduced the interval between the MU stage of microspore development and flowering by 3 days in both Lambada and Spinner barley varieties, consistent with our previous findings in wheat [28]. The HD-induced water deficit hindered the expansion of the peduncles and filaments, leaving the spikes and anthers partially or entirely trapped within the leaf sheaths and bracts. The lodicules, the scale-like organs at the base of the carpels and stamens, were withered and could not open the HD-co-stressed florets via turgor-driven swelling, as observed in almost entirely autogamous barley varieties with atrophied lodicules [50].

According to soil water potential data, Lambada and Spinner plants experienced severe drought stress at a temperature 10 °C above the optimum. Plant RWC has been considered a measure of plant water status, reflecting metabolic activity in tissues [51], and depends on the root water uptake ability driven by transpiration [52]. In our study, compared to the tolerant Lambada variety, HD co-stress had a significant negative effect on the RWC of sensitive Spinner plants. Consistent with our results, a significant HD co-stress-induced reduction in RWC was observed in tomato, maize, *Artemisia*, barley and rice [34,48,53,54].

In our study, HD co-stress did not affect H_2_O_2_ accumulation in Lambada leaves. In contrast to the respective controls, treated Spinner plants exhibited significantly higher H_2_O_2_ levels, indicating that the ROS-scavenging machinery of the sensitive variety was insufficient to protect mesophyll cells from oxidative injury. Our findings support the idea that increased tolerance to different stress combinations is associated with either enhanced antioxidant capacity or reduced ROS buildup [11]. ROS are highly reactive and damage plants by directly attacking membrane lipids, enhancing lipid peroxidation, protein degradation, DNA fragmentation and ultimately cell death [55,56]. Key sources of ROS in photosynthetic tissues include chloroplasts, mitochondria and peroxisomes [57]. ROS participate in signal transduction, but their production increases when plants are exposed to adverse conditions [58], resulting in oxidative stress and plant injury [5]. The HD co-stress-induced increase in H_2_O_2_ content has been reported in studies on chickpeas, *Artemisia* and rice [34,53,59].

Because ROS accumulation differed markedly between the two cultivars, the antioxidant defense system responsible for ROS detoxification was examined. Plants evolved endogenous enzymatic and non-enzymatic defense systems that work together to protect against oxidative damage by scavenging ROS [60], maintaining a balance between ROS production and detoxification through a complex redox network and supporting normal cellular function [38,61]. We assessed the activities of antioxidant enzymes CAT, G-POD, APX, GR, and GST associated with barley plants’ response to combined HD stress. Among control plants of the studied genotypes, we observed genotype-dependent variation in antioxidant enzyme activities: compared with the HD co-stress-tolerant Lambada plants, Spinner exhibited higher total antioxidant enzyme activity. Although G-POD activity declined in Lambada plants under HD co-stress, GST activity increased while CAT, APX, and GR activities remained unchanged. Together, these responses indicate an efficient antioxidant defense mechanism that limits oxidative damage under combined stress. Correspondingly, increased activity of antioxidant enzymes, including CAT, GPX, APX and GR, has been reported in some barley genotypes to alleviate oxidative damage by scavenging H_2_O_2_ [62]. Additionally, CAT and APX are key components of the plant defense system against combined heat and drought stress, owing to their central roles in detoxifying H_2_O_2_ and maintaining cellular redox homeostasis [3,63]. A reduction in enzymatic activities in HD co-stressed Spinner flag leaves indicates that the balance between antioxidant scavenging and ROS production was disrupted, consistent with the sharp decline in antioxidant enzyme activities observed in *Populus yunnanensis* [64]. Overall, Spinner plants accumulated higher levels of ROS and exhibited significantly lower antioxidant enzyme activities. Consistent with our results, an imbalance between ROS production and scavenging negatively affected yield by limiting anther and ovary growth and development, as well as pollen morphology, viability, fertility, and seed set [28,40].

In addition to enzymatic antioxidants, flavonoids also contribute to cellular redox homeostasis. The accumulation of flavonoids under drought stress has been linked to ROS scavenging [65]. The ability of Lambada plants to maintain their flavonoid content during stress could constitute a photoprotective response of the variety, as suggested for citrus [66]. Although an increase in leaf flavonoid content was reported in wild and cultivated barley varieties under drought and salinity stress [67], the total flavonoid content declined in HD co-stressed Spinner plants. These results support our previous findings on the relative resistance of the Lambada cultivar’s photosynthetic pigments to stress-induced degradation, preserved photosynthetic parameters, and leaf ultrastructure [48].

HD co-stress applied for 5 days prior to anthesis resulted in a position-dependent reduction in the sizes of the male and female reproductive organs in both Lambada and Spinner plants. Consistent with our present observations, wheat subjected to heat and drought co-stress during microgametogenesis showed no reduction in anther length or ovary size, but the length of the stylodia was significantly reduced, with the highest rate at the base and top of the spikes [28]. Although HD co-stress seemed to induce proterandry in both genotypes, with significant developmental delay of the pistils, the less-developed stylodia and secondary stigmatic branches were not dysfunctional, as indicated by the fertility data described later. ROS accumulation and, consequently, limitations in the photosynthetic machinery due to stress were reported to reduce the source-to-sink supply of assimilates, cause severe cellular damage, and result in smaller reproductive organs [39,66,68]. Likewise, both heat and drought stresses have been observed to cause morphological and structural anomalies in generative structures [24,69,70,71].

The integrity and internal structure of cells and organelles were better preserved in the male gametophytes of Lambada under HD co-stress. In contrast, stress-induced structural and ultrastructural anomalies were more pronounced in the stress-sensitive Spinner pollen. Significantly shriveled pollen cells were observed in treated anthers of this variety, similar to heat-stress-driven shrinkage of tomato, soybean, wheat, and sorghum pollen [72,73,74,75]. Drought and heat stress are considered to inhibit the enzymatic processes involved in starch synthesis [76,77]. Compared with Lambada male gametophytes, Spinner pollen accumulated less starch, which likely contributed to reduced pollen viability and floret fertility, as starch is the primary energy source for pollen development and germination [70]. HD co-stress during gametogenesis did not alter exine structure in either barley variety, since exine synthesis was already complete by the MU stage of microspore development (the beginning of co-stress treatment). This finding contrasted with the results of Hinojosa et al. [78], who described exine thickening after 7 days of heat stress in quinoa, and Djanaguiraman et al. [75], who reported exine thickening during the flowering stage after high-temperature stress in sorghum. Since intine synthesis begins after the 2nd pollen mitosis, we hypothesized that HD co-stress would significantly affect its development. The intine was reported to be eliminated after water deprivation during meiosis in wheat [79] or thickened as a consequence of heat stress during microsporogenesis in bean [80] and pea [81], or during microgametogenesis in sorghum [75]. Alongside cytoplasmic shrinkage, significant thickening and vacuolization of the inner cell wall were observed in HD co-stressed pollen from both varieties, consistent with previous results [75,81]. Separation of the constricted protoplasmic membrane from the inner cell wall was not observed in HD co-stressed barley pollen, in contrast to drought stress-induced plasmolysis in tomato [82]. HD co-stress during microgametogenesis caused intense vacuolization in the pollen of both barley genotypes. The appearance of double-membrane structures and coiled membranes in the vacuoles of the treated Lambada pollen was a typical feature. Double-membrane structures could serve as reservoirs, facilitating membrane reversion when cells return to normal growth conditions [83]. Swollen mitochondria were observed in heat-and-drought-treated pollen of the sensitive Spinner genotype. These alterations are typical of mitochondria in pre-apoptotic and apoptotic cells [84]. Dilated RER cisternae were observed in treated Spinner pollen cells, similar to those observed during heat-induced dissociation [85]. Compared with Lambada, the number of autophagosomes and autophagic vacuoles in HD co-stressed Spinner pollen was increased. Autophagy, in which cellular components are degraded and nutrients are recycled, is an evolutionarily conserved process that plants continuously maintain throughout their vegetative and generative development to ensure cytoplasmic rearrangements and homeostasis [46,86]. Autophagy is highly associated with the ER, which supplies membranes to form double-membraned autophagosomes, and is upregulated under suboptimal environmental conditions to promote plant viability [87]. An increased number of autophagosomes in Spinner pollen indicates enhanced cellular recycling under HD co-stress. Although autophagy can promote stress tolerance by removing damaged organelles, excessive activation has also been associated with programmed cell death [43,88]. Together with elevated ROS and reduced antioxidant capacity, the pronounced autophagic response in Spinner likely reflects severe cellular stress rather than successful adaptation.

In our study, HD co-stress shortened the duration of tapetum degeneration in anthers and starch accumulation in pollen cells by 3 days in both barley varieties. Proper development of the tapetum, which supplies nutrients and metabolites, is essential for functional pollen [89,90]. Although coordinated degradation is critical for both pollen development and anther dehiscence, abnormal degeneration of this layer can lead to pollen sterility [91]. Thus, the reduction in pollen viability observed in both Lambada and Spinner following HD co-stress treatment is likely associated with premature tapetal degeneration and accelerated pollen development with limited starch accumulation.

Our results are consistent with the findings of Suzuki et al. [85] in snap beans and Fábián et al. [28] in wheat; however, other reports described stress-induced delayed tapetum degeneration [92,93]. Our results are in line with the findings of Iovane and Aronne [94], who reported heat-induced early senescence in tomato pollen.

Fertilization was severely compromised in the main shoots of HD co-stressed plants due to developmental impairment of reproductive organs or gametes. The reduced fertilization rate could be attributed to insufficient starch accumulation in pollen cells and underdeveloped pistils at the time of flowering. Given the presence of tricellular pollen and developmentally arrested female reproductive organs in barley florets at anthesis, it could be assumed that HD co-stress may have accelerated proterandry in both varieties. However, this assumption is contradicted to some extent by the finding of Tímár et al. [95] that the 7-celled female gametophyte forms 3–4 days before tricellular wheat pollen develops. Moreover, stigma receptivity was reported to occur 1–2 days before anthesis in wheat and barley [50,96]. Although the decrease in pollen cell starch content correlated with fertility loss, the severe fertility decline in both varieties also indicated a role for loss of function in the female reproductive organs and gametes. Notably, pistil development negatively correlated with spikelet fertility: compared with treated Spinner plants with more developed pistils, Lambada with shorter secondary stigma branches and smaller ovaries had higher fertilization rates and grain production. Our results are consistent with the findings of Backhaus et al. [97], who reported that large differences in fertility occur within a cereal spike, with the central spikelets producing the highest number of grains. Slafer et al. [98] reported that the number of basal florets and spikelets decreased under shading due to reduced photosynthetic activity and, consequently, resource availability. Delayed development of basal florets explains their abortion in the spike [97]. We suggest that the severe reduction in spikelet fertility and grain production in the basal and top florets of the main spikes might be attributed not only to hierarchical resource allocation to developing ovaries, as suggested by Shen et al. [99], but also to the scarcity of assimilates available for developing anthers during microgametogenesis. The extent of stress-induced reductions in fertility and grain production across culms depended heavily on the genotype and on the developmental stage at which the MMCs, microspores or pollen cells encountered the stress. In Lambada plants, main shoots with MU microspores were the most sensitive to HD co-stress. In contrast, first and second tillers of Spinner plants with diads and tetrads in their anthers showed the greatest reductions in both parameters. Low fertility and grain production in tillers jointing during and booting after HD co-stress might be attributed to reduced photosynthesis and senescence due to the 5-day co-stress [48], and to competition for assimilates between main shoots and tillers and between spikes and stems [100,101]. The mechanisms underlying the differential tolerance of the barley cultivars Lambada and Spinner are described in Figure 10.

## 4. Conclusions and Future Perspectives

Overall, our results indicate that the greater tolerance of the Lambada genotype is primarily attributable to its superior ability to maintain cellular redox homeostasis under combined heat and drought stress. The maintenance of antioxidant enzyme activities, together with stable flavonoid levels, prevented excessive H_2_O_2_ accumulation in the flag leaves despite severe water deficit. Combined with the observed higher relative water content, these responses likely preserved photosynthetic function and the supply of assimilates to developing reproductive organs. Consequently, oxidative damage in reproductive tissues was reduced, as reflected by better preservation of pollen ultrastructure, greater starch accumulation, lower autophagic activity, and ultimately higher spikelet fertility and grain production. These findings suggest that antioxidant defense does not act in isolation but constitutes a central component of an integrated physiological mechanism linking flag-leaf function with reproductive resilience under combined heat and drought stress.

Further studies are needed to determine the basis for the severe, HD co-stress-induced reduction in fertility of the barley main spikes. The role of tapetum malfunction in reduced pollen viability and pollen function needs to be clarified. Reciprocal crosses (control pollen × stressed pistils) and comparative histological examinations of pistils are necessary to determine how and to what extent developmental delay and reduced functionality of female reproductive organs and cells contribute to the fertility deficit in barley.

## 5. Materials and Methods

### 5.1. Plant Material, Experimental Design and Stress Conditions

Plants of the two-rowed winter barley (*Hordeum vulgare* L.) genotypes, Lambada (Regina × Rifle, RAGT UK, 200) and Spinner (G84095-3043 × Velvet, Lantmännen, Sweden, 1999), were used in this study. Based on our earlier research [48], Lambada and Spinner plants were identified as HD co-stress tolerant and HD co-stress sensitive, respectively. After 8 weeks of vernalization at 4 °C, individual plants were grown in pots (n = 100 per genotype and treatment) containing 2 kg of a soil-sand-peat mixture (3:1:1, *v*/*v*/*v*) and cultivated in growth chambers (Conviron, Winnipeg, MB, Canada) using the T1 spring climatic program [102] under optimal growth conditions until the mid-uninucleate (MU) stage of microspore development in the main shoot florets. Throughout plant development under optimal conditions, irrigation was carried out regularly in the morning at a rate of 150 mL/day. The relative daily min/max air humidity was 50%/75%. The daily maximum photosynthetic photon flux of 300 μmol m^−2^ s^−1^ was used. The min/max temperature was 10 °C/20 °C, and the photoperiod was 15 h under control conditions during microgametogenesis. The circadian rhythm of the environmental conditions in control growth chambers during microgametogenesis is provided in Supplementary Table S1. During the experiment, the plants were randomly rearranged each week within the growth chambers to minimize border effects caused by variations in light and temperature. The MU stage of the microspores in the spikes of the main shoots was identified by acetocarmine staining (1% Carmine in 45% acetic acid) followed by light microscopy. The distance between the flag leaf and penultimate leaf auricles at the MU stage of the main shoot was recorded (n = 5), and subsequent plant selection was based on this distance (8–10 mm in both genotypes). Alongside the main shoots, the developmental stage of male gametes in all tillers (up to a maximum of 4) that had emerged by that time was recorded. Plants at the appropriate developmental stage were transferred to stress chambers. HD co-stress was induced by total water deprivation at the min/max night/day temperatures of 20 °C/30 °C from the MU stage through Pollen Mitosis I and II in the main shoots under controlled conditions. The daily min/max relative air humidity was 30%/65% during HD co-stress. The circadian rhythm of the environmental conditions during treatment is described in Supplementary Table S2. The end of the 5-day treatment coincided exactly with anther dehiscence in the main spikes (Z60) in both genotypes across all 3 independent biological replicates [103]. The days from the MU stage to flowering were recorded for each plant. The pistils, anthers and microspores were sampled 2 h before the onset of anthesis (9:00 am on the day of flowering in the main spikes) in at least 3 biological and 3 technical repetitions per genotype and treatment. Flag leaves of the main shoots were collected at 11 am at the end of the five-day treatment (anthesis) for measurements and assays, with at least 3 biological and 3 technical repetitions per genotype and treatment. For each genotype and treatment, 15 plants were re-irrigated and grown to full maturity under control conditions, with a final min/max night/day temperature of 24/32 °C. The experimental timeline and workflow are presented in Figure 11.

### 5.2. Water Status Measurements

The volumetric water content (VWC) of the soil (n = 10 pots per genotype and treatment) was monitored using an HH2 moisture meter connected to an SM200 soil water sensor (Delta-T Devices Ltd., Cambridge, UK) by measuring changes in the apparent dielectric constant at full water saturation and during the treatments. The relative water content (RWC) of the flag leaves (n = 15 per genotype and treatment) was determined at the end of HD co-stress using the following equation: $RWC\;\left(\%\right)=\frac{FW-DW}{SW-DW}\times 100$ [104], where FW is the fresh weight at sampling, SW is the saturated weight after 24 h of rehydration with distilled water at 25 °C in the dark, and DW is the dry weight after drying for 24 h at 80 °C.

### 5.3. Hydrogen Peroxide Detection and Quantification

The subcellular localization of H_2_O_2_ was detected through the formation of a deep brown polymerization product upon its reaction with 3,3-diaminobenzidine (DAB) [105]. The DAB assay was performed on flag leaves (n = 9 per genotype and treatment). Sampling was performed using three consecutive rectangular areas (1 cm long each) on one side of each leaf blade. The segments extended from the leaf margin toward the midrib, which was excluded from the sampled tissue. The samples were immersed in a DAB solution (1 mg mL^−1^), vacuum infiltrated for 10 min, incubated overnight at room temperature (RT), washed with MQ water, cleared with 100% ethanol, and examined under a BX5 light microscope (Olympus, Tokyo, Japan). The H_2_O_2_ content of the flag leaves (1 cm, n = 9 per genotype and treatment) was measured by observing the peroxide-mediated oxidation of ferrous ions and the subsequent reaction of ferric ions with xylenol orange (FOX1 assay) [106]. The absorbance of the Fe^3+^-xylenol orange complex was measured photometrically (UV–VIS 160A, Shimadzu Corp., Kyoto, Japan) at 560 nm and expressed as µM of H_2_O_2_ g^−1^, DW based on a calibration curve.

### 5.4. Analysis of Antioxidant Enzyme Activities

For the analysis of antioxidant enzyme activities, 500 mg of leaf tissue (n = 15 per genotype and treatment) was homogenized in 2.5 mL of ice-cold Tris-HCl buffer (0.5 M, pH 7.5), which contained 3 mM MgCl_2_ and 1 mM EDTA. Catalase (CAT) activity of the extract was measured spectrophotometrically by monitoring the decrease in absorbance at 240 nm [107]. The reaction mixture consisted of 0.44 M Tris-HCl buffer (pH 7.4), 10 mM H_2_O_2_, and enzyme extract. Guaiacol peroxidase (G-POD) activity was measured at 470 nm as described by Ádám et al. [108]. The reaction mixture consisted of 88 mM Na-acetate buffer (pH 5.5), 0.88 mM guaiacol, 10 mM H_2_O_2_, and the enzyme extract. Ascorbate peroxidase (APX) activity of the extract was determined in the presence of 0.2 M Tris-HCl buffer (pH 7.8) and 5.625 mM ascorbic acid. The reaction was initiated with 0.5 mM H_2_O_2_, and the decrease in absorbance at 290 nm was monitored as described by Janda et al. [107]. Glutathione reductase (GR) activity was determined at 412 nm, according to Smith et al. [109]. The reaction mixture contained 75 mM Na-phosphate buffer (pH 7.5), 0.2 mM diethylenetriamine-pentaacetic acid, 0.75 mM 5,5′-dithiobis-(2-nitrobenzoic acid), 0.1 mM NADPH, 0.5 mM oxidized glutathione, and 50 μL of plant extract in a total volume of 1 mL. Glutathione-S-transferase (GST) activity was measured by monitoring changes in absorbance at 340 nm in a mixture containing 72.7 mM Na-phosphate buffer (pH 6.5), 3.6 mM reduced glutathione, 1 mM 1-chloro-2,4-dinitrobenzene, and enzyme extract [110]. Enzymatic activities were determined photometrically (UV–VIS 160A, Shimadzu Corp., Kyoto, Japan) and are expressed in nkatal g^−1^ DW (nkatal corresponds to the enzymatic activity that catalyzes the transformation of one nmole of substrate per second).

### 5.5. Total Flavonoid Content

Total flavonoid content (TFC) was determined as described by Zhishen et al. [111] with modifications. Flag leaves (200 mg; n = 15 per genotype and treatment) were homogenized in 1 mL of 60% ethanol. Ethanol (60%) was added to a total volume of 2 mL, and the mixture was incubated with 0.25 mL of 5% (*w*/*v*) NaNO_2_. After 6 min, 0.25 mL of 10% (*w*/*v*) AlCl_3_ and 2 mL of 1 M NaOH were added to the reaction mixture, and the total volume was adjusted to 8 mL with 60% ethanol. The TFC was measured spectrophotometrically (UV–VIS 160A, Shimadzu Corp., Kyoto, Japan) at 510 nm and expressed as milligrams of quercetin equivalent (QE) g^−1^ DW based on a calibration curve.

### 5.6. Morphometric Analysis of Pistils and Anthers

Pistils and anthers from the basal, central, and top florets were collected from 3 main spikes per genotype and treatment. To prevent anther dehiscence, the organs were isolated on the morning of anthesis (the 5th day of treatment). Rudimentary florets at the base and top of the spikes were not excluded. Micrographs were captured with a Discovery V8 stereomicroscope (Zeiss, Darmstadt, Germany) equipped with an HD Ultra camera (Euromex, Arnhem, The Netherlands). Anther length, ovary length, ovary width, and stigma length were measured using Image-Pro Plus 7.0 software (Media Cybernetics, Rockville, MD, USA).

### 5.7. Light Microscopy

For structural studies, to prevent dehiscence, anthers were isolated from the central region of the spikes on the morning of anthesis (the 5th day of treatment). The anthers were fixed in 60 mM Sörensen’s buffer (SB; pH 7.2) containing 4% formaldehyde (*w*/*v*) overnight, washed with SB, dehydrated through a gradient series of ethanol, and infiltrated with Unicryl resin (BBI International, Grand Forks, ND, USA) according to the manufacturer’s instructions. The resin was polymerized by ultraviolet light at −20 °C for 48 h. Semi-thin (1 µm) sections were cut at the longitudinal plane of the anthers using an Ultracut-E microtome (Reichert-Jung, Heidelberg, Germany). The sections were stained with 1% periodic acid-Schiff (PAS) for polysaccharides and 1% Amido black 10B for proteins. The stained sections were mounted in glycerol and examined with a BX51 light microscope (Olympus, Tokyo, Japan). For morphological analysis of pollen, anthers from control and HD co-stressed plants (n = 9 per genotype, treatment, and location) were sampled from three regions of the spikes at anthesis (base, center, and top). The anthers were chopped on glass slides with a scalpel, releasing pollen into a drop of 1% carmine in Farmer’s fluid (1:3 glacial acetic acid:100% ethanol). Anther debris was removed, the drop was covered with a coverslip, and the pollen was examined under a BX5 light microscope (Olympus, Tokyo, Japan) equipped with an HD Ultra camera (Euromex, Arnhem, The Netherlands).

### 5.8. Transmission Electron Microscopy

Anthers (n = 9 per genotype and treatment) were isolated from the central region of the spikes and fixed in 2.5% (*v*/*v*) glutaraldehyde in 60 mM SB, as described above. Samples were postfixed with 1% OsO_4_ for 4 h, dehydrated through an ascending ethanol series, and embedded in Durcupan ACM resin (Merck, Darmstadt, Germany) according to the manufacturer’s instructions. Ultrathin sections (70 nm) were cut using an Ultracut-E microtome (Reichert-Jung, Heidelberg, Germany), mounted on 100-mesh nickel grids coated with Formvar (SPI-Chem, West Chester, PA, USA), and examined with a transmission electron microscope (Hitachi 7100, Hitachi Ltd. Tokyo, Japan) operating at 80 keV. The thicknesses of the exine and intine in pollen cells, located in the central region of the main spikes, were measured with Image-Pro Plus 7.0 software (Media Cybernetics, Rockville, MD, USA).

### 5.9. Pollen Viability Assay

Pollen viability of the main spikes was assessed using the method described by Trognitz [112], with modifications. Barley pollen collected separately from the base, middle, and top regions of the spikes (n = 9 per genotype, location, and treatment) was incubated in 0.06 M Sörensen’s buffer (pH 7.2) containing 0.5% 2,3,5-triphenyltetrazolium chloride (TTC) for 15 min in the dark at 37 °C. In this assay, viable pollen converts the colorless TTC to red formazan within pollen cells via dehydrogenases. Pollen viability was calculated as the percentage of colored pollen relative to the total number of male gametophytes observed across 15 microscopic fields of view. A minimum of 2218 pollen grains were evaluated per genotype and treatment.

### 5.10. Assessment of Yield Constituents for Individual Culms and Plants

Control and HD co-stressed plants (n = 15 per genotype and treatment) were harvested and hand-threshed at full maturity. The number of culms, productive culms, spikelets and grains per spike region (base, center, and top) was counted, and the grains were weighed. The fertility indices of florets in different regions were determined as the ratio of potential to actual caryopsis number, with three grains per spikelet used to calculate the potential grain number.

### 5.11. Statistical Analysis

All data were pooled means from at least 3 biological and 3 technical replicates per genotype and treatment. Data were analyzed using ANOVA (SPSS for Windows, version 16.0, IBM Corp., Armonk, NY, USA). Mean values were compared using Tukey’s multiple range test, with significance set at *p* ≤ 0.05. Means and standard deviations are presented in tables and figures. Pearson’s correlation coefficient was used to deduce the relationships between the measured traits (SSPS for Windows, version 16.0, IBM Corp., Armonk, NY, USA). Correlation coefficients (r) were interpreted according to Schober et al. [113]: 1–0.9 absolute magnitude (AM) of very strong correlation, 0.89–0.7 AM of strong correlation, 0.69–0.4 AM of moderate correlation, 0.39–0.1 AM of weak correlation, 0.1–0 AM of negligible correlation. Mean values and standard deviations are presented in tables and figures. The standard deviation (SD) of means and differences between treatments were compared pairwise at a *p* ≤ 0.05 level of probability.

## Figures and Tables

**Figure 1:**
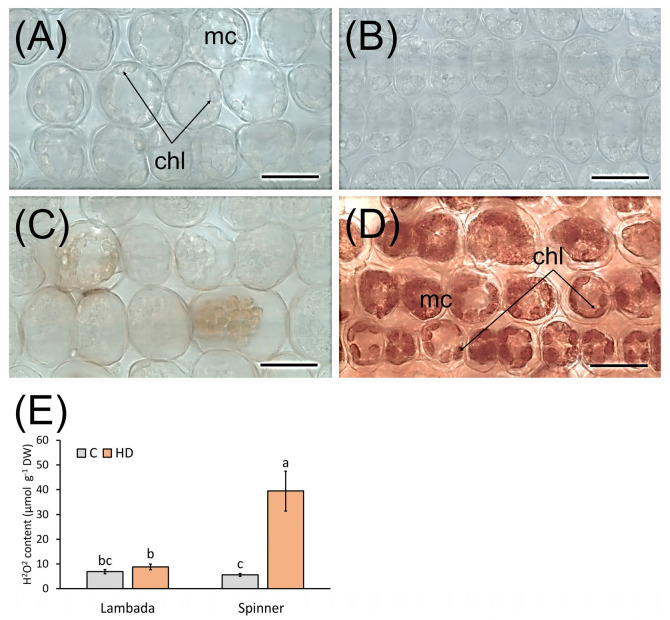
Localization of H_2_O_2_ in the mesophyll cells of control (**A**,**B**) and heat-and-drought co-stressed (**C**,**D**) Lambada (**A**,**C**) and Spinner (**B**,**D**) plants and H_2_O_2_ content of barley flag leaves ((**E**) representative images). mc: mesophyll cell; chl: chloroplast; brown coloration: H_2_O_2_ positivity. Mean values along with standard deviations are presented. Different letters within columns indicate significant differences between means at the *p* ≤ 0.05 level of probability. Bar = 20 µm.

**Figure 2:**
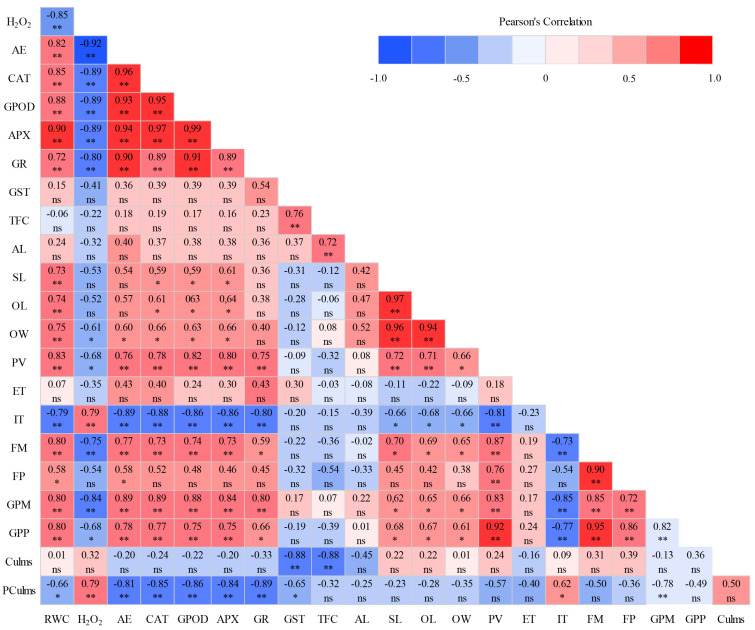
Pearson’s correlation coefficients (r) describing associations of physiological, biochemical, morphological and agronomical traits. AE: total antioxidant enzyme activity; CAT: catalase activity; G-POD: guaiacol peroxidase activity; APX: ascorbate peroxidase activity; GR: glutathione reductase activity; GST: glutathione-S-transferase activity; TFC: total flavonoid content; AL: anther length; SL: stigma length; OL: ovary length; OW: ovary width; PV: pollen viability; ET: exine thickness; IT: intine thickness; FM: main shoot fertility; FP: plant fertility; GPM: grain production of the main shoot; GPP: plant grain production; Culms: number of culms at ripening; PCulms: productive culms at ripening. 1–0.9 (absolute magnitude (AM) of very strong correlation), 0.89–0.7 (AM of strong correlation), 0.69–0.4 (AM of moderate correlation), 0.39–0.1 (AM of weak correlation), 0.1–0 (AM of negligible correlation). ns not significant; * correlation is significant at the 0.05 level of probability (two-tailed Pearson’s correlation test); ** correlation is significant at the 0.01 level of probability (two-tailed Pearson’s correlation test).

**Figure 3:**
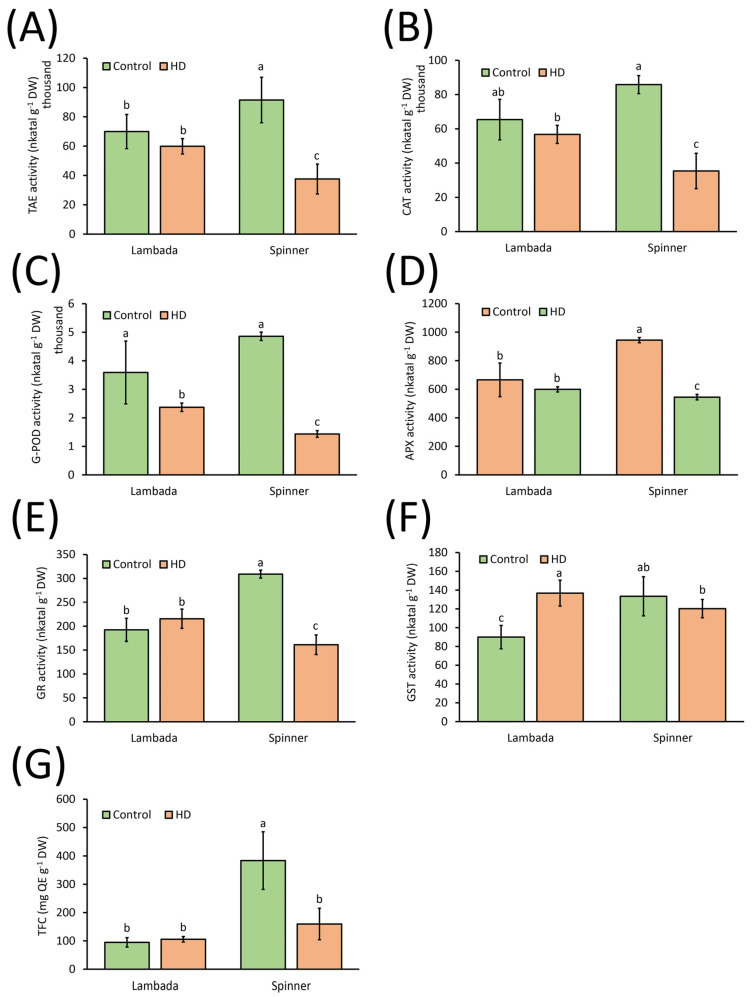
Antioxidant enzyme activities (**A**–**F**) and total flavonoid content (**G**) in control and heat and drought co-stressed barley flag leaves. APX: ascorbate peroxidase; CAT: catalase; DW: dry weight; G-POD: guaiacol peroxidase; GR: glutathione reductase; GST: glutathione-S-transferase; HD: heat and drought co-stress; TAE: total antioxidant enzyme; TFC: total flavonoid content. Mean values along with standard deviations are presented. In each histogram, different letters above columns indicate significant differences between means at the *p* ≤ 0.05 level of probability.

**Figure 4:**
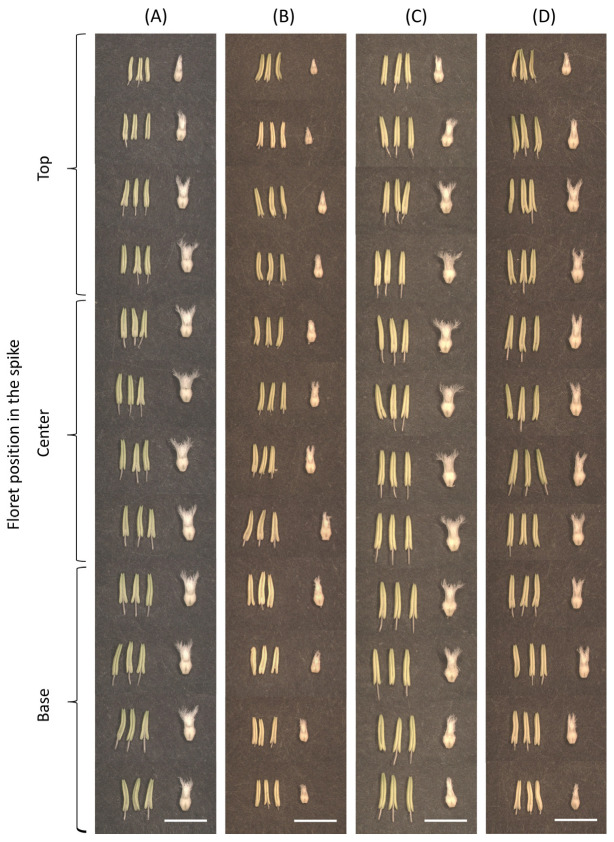
Location-dependent morphology of control (**A**,**C**) and heat and drought co-stressed (**B**,**D**) Lambada (**A**,**B**) and Spinner anthers and pistils (**C**,**D**) prior to anthesis (representative images). Note that compared with the anthers, pistil development in heat and drought co-stressed florets was delayed. Bar = 6 mm.

**Figure 5:**
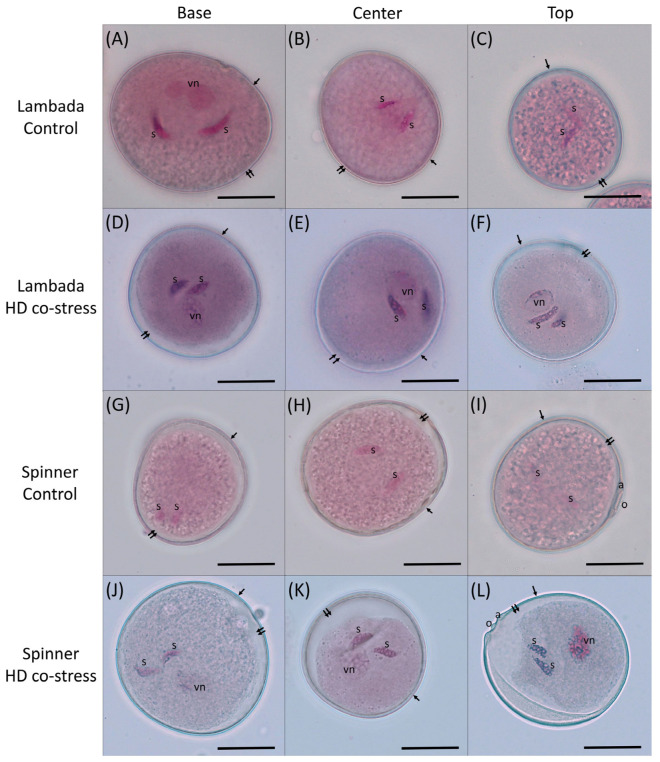
Morphology of control (**A**–**C**,**G**–**I**) and heat and drought co-stressed (**D**–**F**,**J**–**L**) Lambada (**A**–**F**) and Spinner (**G**–**L**) winter barley pollen isolated from the basal (**A**,**D**,**G**,**J**), central (**B**,**E**,**H**,**K**) and top (**C**,**F**,**I**,**L**) regions of the main spikes at anthesis (representative images). Note that, irrespective of genotype and treatment, the vast majority of pollen cells were tricellular with spindle-like sperm cells at anthesis. Compared with the extended starch reserves present in control pollen, fewer starch granules accumulated in heat and drought co-stressed male gametophytes isolated from the top (**F**) of Lambada and from all positions of Spinner spikes (**J**–**L**). Due to the high level of starch deposition, the visibility of vegetative nuclei was limited in control pollen. a: aperture; s: sperm cell; sg: starch granule; o: operculum; vn: vegetative nucleus; arrow: exine; double arrow: intine. Bar = 30 µm.

**Figure 6:**
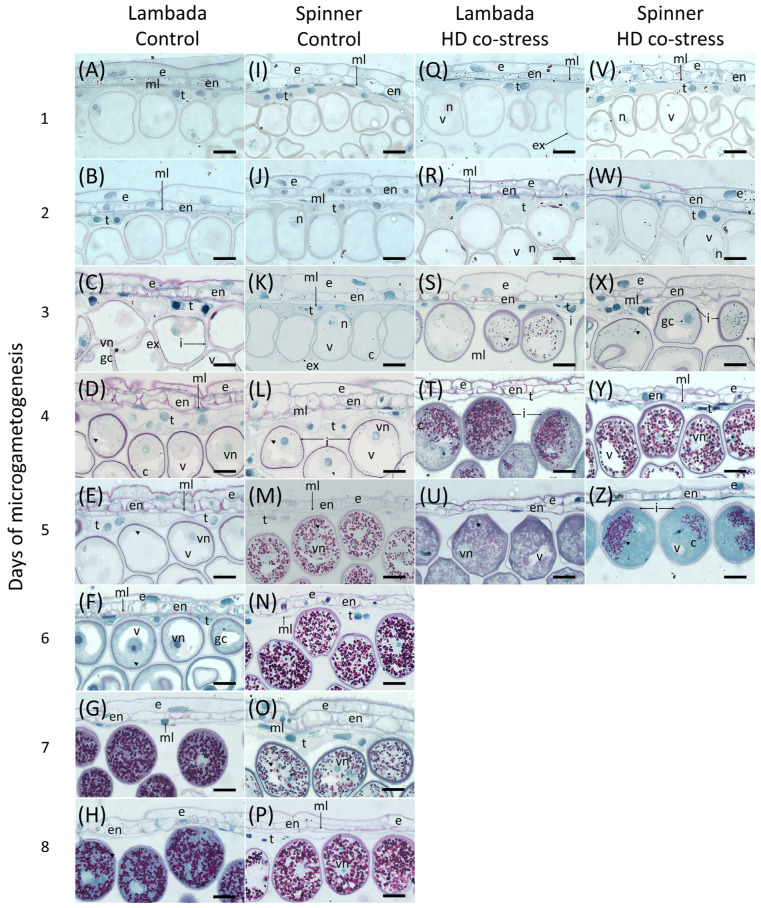
Time order of microgametogenesis and tapetum degradation in the anthers of control (**A**–**P**) and heat-and-drought-treated (**Q**–**Z**) plants (representative images). c: cytoplasm; e: epidermis; en: endothecium; ex: exine; gc: generative cell; i: intine; ml: middle layer; n: nucleus of a uninucleate microspore; t: tapetum; v: vacuole; vn: vegetative nucleus; arrowhead: starch granule; asterisk: sperm cell. Blue coloration: proteins; magenta coloration: carbohydrates. Bar = 15 μm.

**Figure 7:**
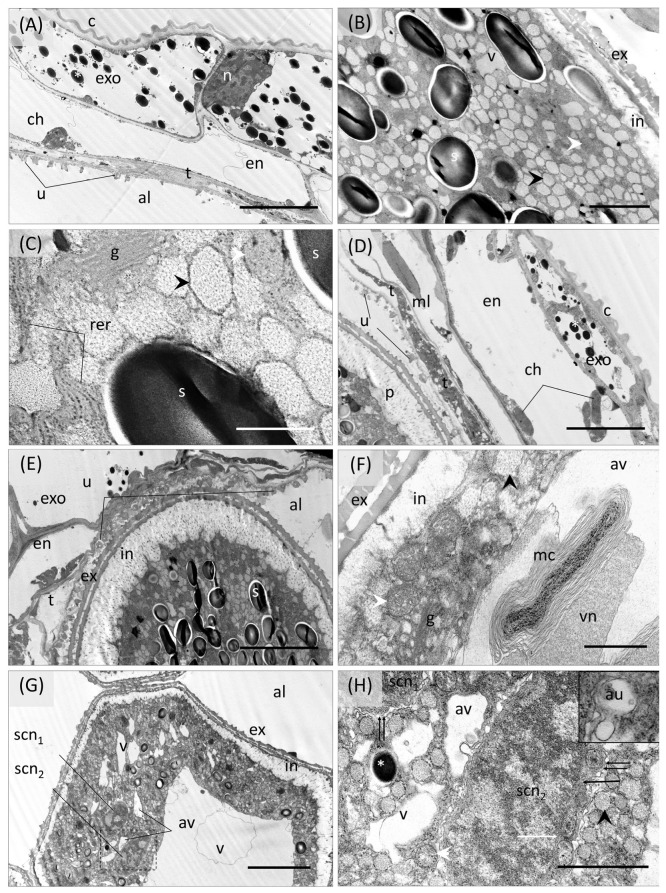
Ultrastructure of control (**A**–**C**) and heat and drought co-stressed (**D**–**H**) Lambada (**A**–**F**) and Spinner (**G**,**H**) anthers (**A**,**D**) and pollen cells (**B**,**C**,**E**–**H**) at anthesis (representative images). af: autophagosome; al: anther lumen; av: autophagic vacuole; c: cuticle; ch: chloroplast; exo: exothecium; en: endothecium; end: endexine; ex: exine; g: Golgi-apparatus; in: intine; mc: membrane coiling; ml: middle layer; n: nucleus; p: pollen; rer: rough endoplasmic reticulum; s: starch; scn1; scn2: sperm cell nuclei; t: tapetum; u: Ubisch bodies/orbicules; v: vacuole; vn: vegetative nucleus; asterisk: lipid body; black arrowhead: Golgi vesicle; black arrow: sperm cell cytoplasm; double black arrow: plasmamembrane; white arrow: nuclear envelope; white arrowhead: mitochondrium. Bar = 5 µm (**A**,**B**,**E**); 2 µm (**C**,**H**); 500 nm (**D**); 1 µm (**F**); 10 µm (**G**).

**Figure 8:**
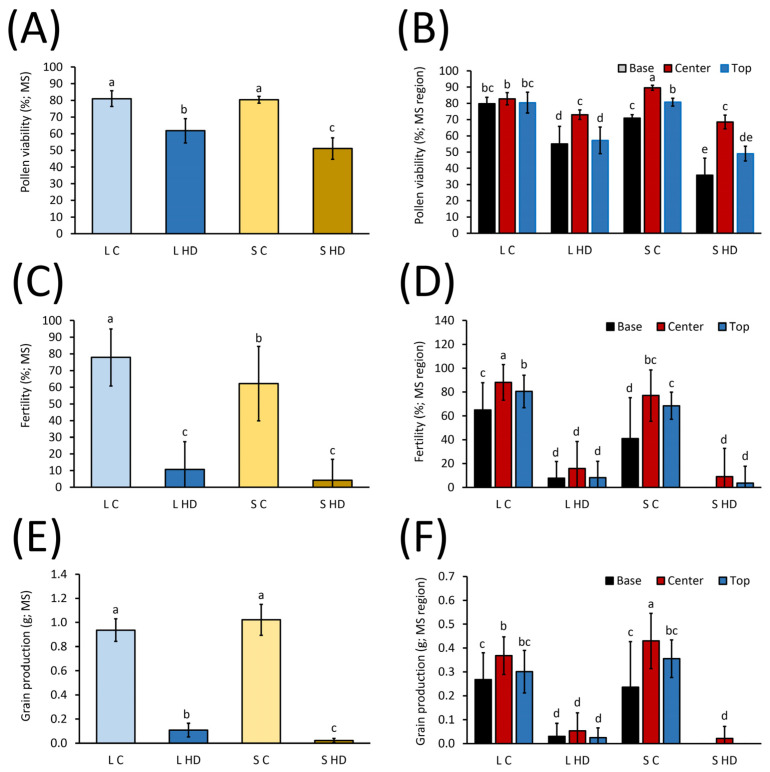
Pollen viability (**A**,**B**), floret fertility (**C**,**D**) and production (**E**,**F**) of control and heat and drought co-stressed main spikes (**A**,**C**,**E**) and spike regions (**B**,**D**,**F**). C: control; HD: heat and drought co-stress; MS: main spike; L: Lambada; S: Spinner. Mean values and standard deviations are presented. In each histogram, different letters above the columns indicate significant differences between means at the *p* ≤ 0.05 level of probability.

**Figure 9:**
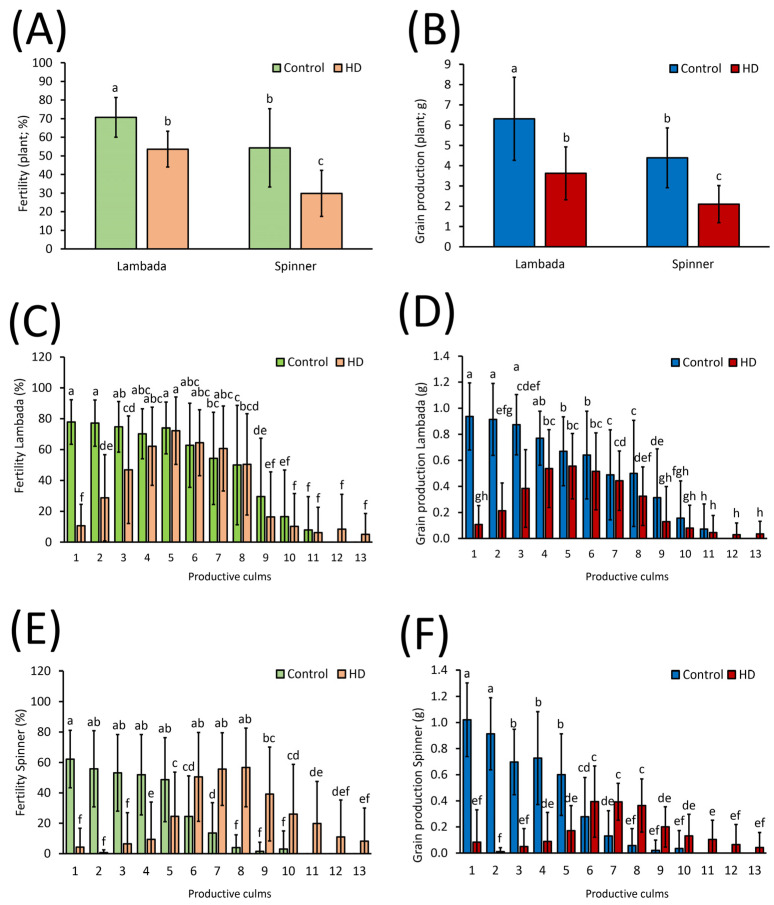
Fertility ratio (**A**,**C**,**E**) and grain production (**B**,**D**,**F**) of Lambada (**A**–**D**) and Spinner (**A**,**B**,**E**,**F**) plants (**A**,**B**) and individual (1–13) productive culms (**C**–**F**). 1 (main shoot), 2–13 (tillers), HD: heat and drought co-stress. Mean values, along with standard deviations, are presented. In each histogram, different letters above the columns indicate significant differences between means at the *p* ≤ 0.05 level of probability.

**Figure 10:**
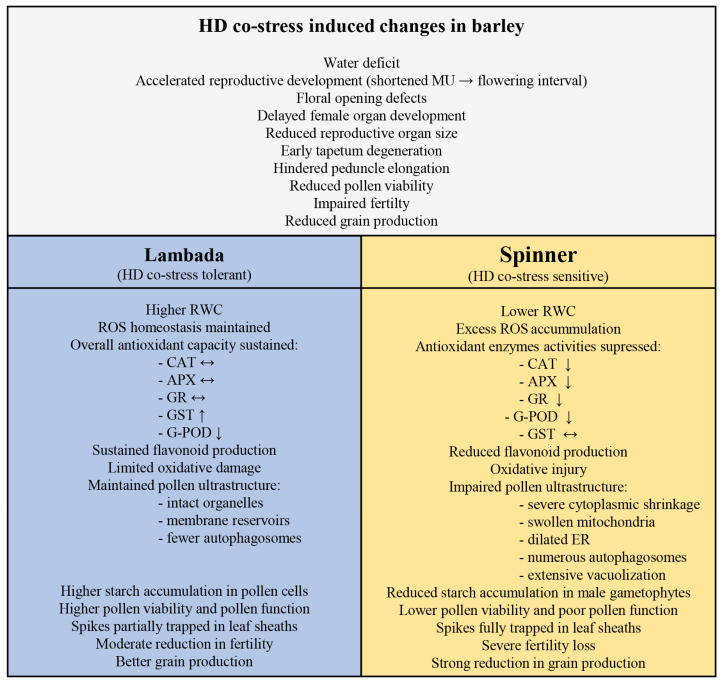
Proposed schematic model illustrating combined heat- and drought-induced changes in barley and the mechanisms underlying the differential tolerance of the barley cultivars Lambada and Spinner to stress during microgametogenesis. APX: ascorbate peroxidase activity; CAT: catalase activity; ER: endoplasmic reticulum; G-POD: guaiacol peroxidase activity; GR: glutathione reductase activity; GST: glutathione-S-transferase activity; HD: heat and drought; MU: mud-uninucleate; ROS: reactive oxygen species.

**Figure 11:**
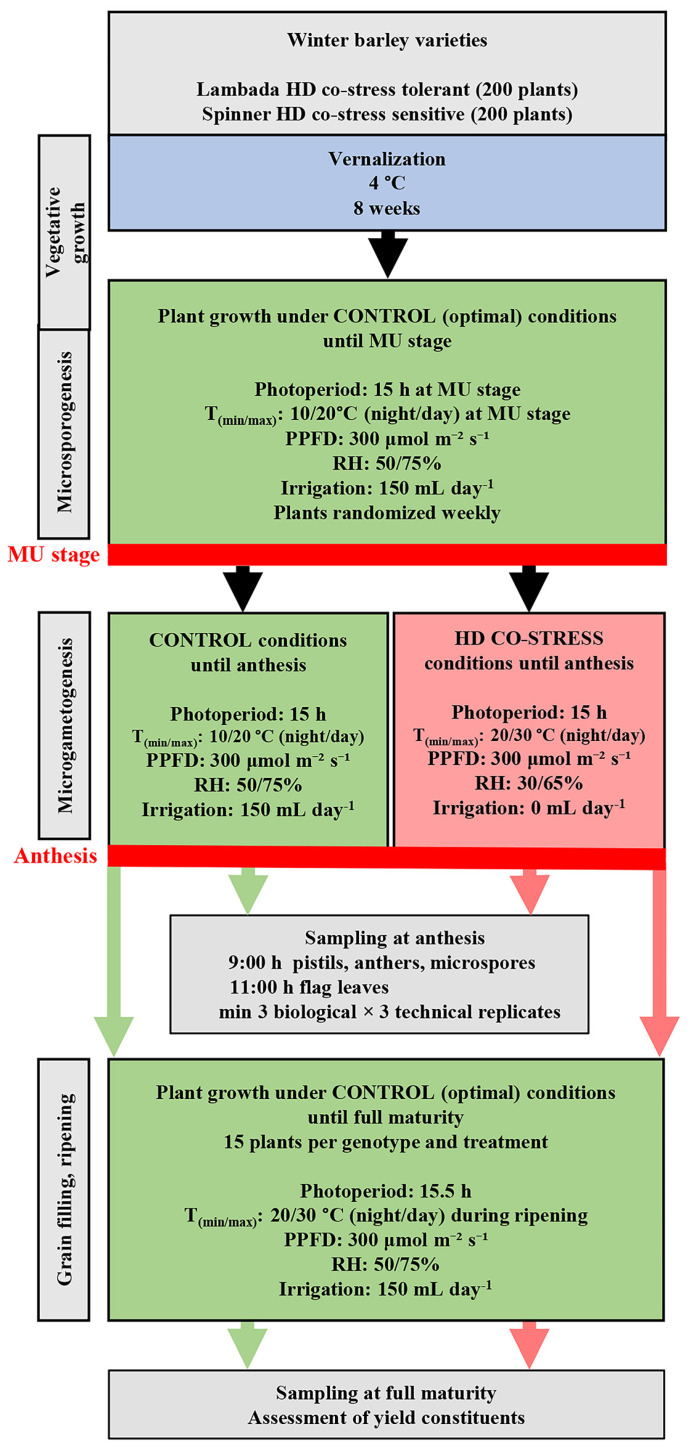
Experimental timeline and workflow for heat and drought co-stress during barley gametogenesis. HD: heat and drought; MU: mid-uninucleate; PPFD: photosynthetic photon flux density; RH: relative humidity; T_(min/max)_: minimum/maximum temperature.

**Table 1:** Number of culms and spikes in Lambada and Spinner plants at anthesis and at ripening.

Trait (No.)	Lambada		Spinner	
	Control	HD	Control	HD
Culms at anthesis	7.9 ± 1.0 ^a^	7.5 ± 0.5 ^a^	7.9 ± 0.9 ^a^	6.8 ± 1.0 ^b^
Emerged spikes at anthesis	2.3 ± 0.7 ^a^	1.4 ± 0.6 ^b^	2.1 ± 0.8 ^a^	0.5 ± 0.5 ^c^
Culms at ripening	14.3 ± 2.1 ^a^	13.5 ± 1.6 ^a^	11.9 ± 1.6 ^b^	13.2 ± 1.7 ^ab^
Productive culms at ripening	8.4 ± 1.7 ^a^	8.4 ± 2.2 ^a^	6.2 ± 1.9 ^b^	9.9 ± 1.8 ^a^

Values represent means ± standard deviations. HD: heat and drought co-stress. Means in each row with different superscript letters are significantly different at *p* ≤ 0.05 level of probability.

**Table 2:** Relative water content of control and heat and drought co-stressed Lambada and Spinner flag leaves at anthesis.

Genotype	RWC (%)	
	Control	HD Co-Stress
Lambada	72.1 ± 4.5 ^a^	60.6 ± 4.3 ^b^
Spinner	77.5 ± 4.6 ^a^	46.9 ± 4.7 ^c^

Values represent means ± standard deviations. Means superscripted by different letters are significantly different at the *p* ≤ 0.05 level of probability. RWC: relative water content, HD: heat and drought.

**Table 3:** Size of reproductive organs in control and HD co-stressed florets of Lambada and Spinner varieties on the morning of anthesis.

		Lambada		Spinner	
Parameter (mm)	Location in the Spike	Control	HD Co-Stress	Control	HD Co-Stress
Anther length	Top	4.75 ± 0.57 ^cd^	4.13 ± 0.42 ^e^	5.52 ± 0.37 ^a^	5.24 ± 0.37 ^b^
	Middle	4.90 ± 0.19 ^c^	4.45 ± 0.35 ^d^	5.60 ± 0.29 ^a^	5.19 ± 0.26 ^b^
	Base	4.84 ± 0.26 ^c^	3.76 ± 0.49 ^e^	5.37 ± 0.31 ^b^	4.70 ± 0.34 ^cd^
Stigma Length	Top	3.58 ± 0.44 ^ab^	1.91 ± 0.56 ^d^	3.24 ± 0.48 ^b^	2.95 ± 0.37 ^bc^
	Middle	3.80 ± 0.12 ^a^	2.53 ± 0.33 ^c^	3.78 ± 0.26 ^a^	3.20 ± 0.25 ^b^
	Base	3.72 ± 0.17 ^a^	1.75 ± 0.43 ^d^	3.29 ± 0.27 ^b^	2.77 ± 0.26 ^bc^
Ovary length	Top	1.91 ± 0.33 ^ab^	1.10 ± 0.22 ^d^	1.70 ± 0.15 ^b^	1.59 ± 0.21 ^bc^
	Middle	2.10 ± 0.19 ^a^	1.43 ± 0.22 ^c^	1.99 ± 0.20 ^a^	1.76 ± 0.18 ^b^
	Base	1.95 ± 0.16 ^ab^	1.09 ± 0.18 ^d^	1.77 ± 0.17 ^b^	1.58 ± 0.28 ^bc^
Ovary width	Top	1.80 ± 0.33 ^b^	1.26 ± 0.20 ^d^	1.77 ± 0.13 ^b^	1.67 ± 0.16 ^b^
	Middle	2.04 ± 0.18 ^a^	1.52 ± 0.17 ^c^	2.05 ± 0.23 ^a^	1.77 ± 0.11 ^b^
	Base	1.86 ± 0.11 ^ab^	1.15 ± 0.19 ^d^	1.74 ± 0.16 ^b^	1.55 ± 0.12 ^bc^

Values represent means ± standard deviations. For each parameter, means with different superscripts are significantly different at the *p* ≤ 0.05 probability level. HD: heat and drought.

## Data Availability

The data supporting the findings of this study are available from the corresponding author upon reasonable request.

## Supplementary Materials

The following supporting information can be downloaded at: https://www.mdpi.com/article/10.3390/plants15152360/s1. Table S1. Circadian changes in optimal (control) environmental conditions during gametogenesis. Table S2. Daily changes in environmental conditions during HD co-stress.

## Abbreviations

APX	ascorbate peroxidase
CAT	catalase
DAB	3,3-diaminobenzidine
DAMU	day after the mid-uninucleate stage of microspore development
DW	dry weight
FW	fresh weight
G-POD	guaiacol peroxidase
GR	glutathione reductase
GST	glutathione-S-transferase
H_2_O_2_	hydrogen peroxide
HD	heat and drought
FOX1	ferrous oxidation xylenol orange, version 1
MMC	microspore mother cell
MU	mid-uninucleate
PAS	periodic acid-Schiff
ROS	reactive oxygen species
RT	room temperature
RWC	relative water content
SB	Sörensen’s buffer
SW	saturated weight
TBARS	2-thiobarbituric acid reactive substances
TGW	thousand-grain weight
TFC	total flavonoid content
TTC	2,3,5-triphenyltetrazolium chloride
VWC	volumetric water content
