# Supplementary material for: Heat and Drought Co-Stress During Microgametogenesis Altered Pollen Development, Fertility and Grain Production in Two-Rowed Winter Barley
Source: Plants (Basel). 2026 Jul 31;15(15):2360. doi: 10.3390/plants15152360 (PMC13467959; doi:10.3390/plants15152360)
Supplement: Supplementary file 1 [file plants-15-02360-s001.zip › plants-4442498-supplementary.pdf]

SUPPLEMENTARY MATERIAL

Heat and drought co-stress during microgametogenesis altered pollen development, fertility and grain production in two-rowed winter barley.

Katalin Jäger, Emmanuel A. Jampoh, Dorina Babinyec-Czifra, Zoltán Kristóf & Beáta Barnabás

Supplementary Table S1. Circadian changes in optimal (control) environmental conditions during gametogenesis.

Time	Temperature (°C)	Relative humidity (%)	PPFD (µmol m^{-2} s^{-1})
0:00	11	75	0
2:00	11	75	0
3:45	10	75	0
5:00	10	75	65
6:00	12	70	150
6:30	12	70	206
7:00	16	65	206
8:00	17	65	206
9:30	18	60	300
10:00	19	55	300
11:00	20	50	300
13:00	20	50	300
14:30	20	50	206
16:00	19	55	206
17:00	18	55	206
18:00	17	60	150
19:30	16	65	150
20:00	15	65	65
21:00	14	70	0
22:00	12	70	0
23:00	11	70	0

PPFD: photosynthetic photon flux density

Supplementary Table S2. Daily changes in environmental conditions during HD co-stress.

Time	Temperature (°C)	Relative humidity (%)	PPFD (μmol m^{-2} s^{-1})
0:00	21	65	0
2:00	21	65	0
3:45	20	65	0
5:00	20	60	65
6:00	22	60	150
6:30	22	55	206
7:00	26	50	206
8:00	27	45	206
9:30	28	40	300
10:00	29	35	300
11:00	30	30	300
13:00	30	30	300
14:30	30	30	206
16:00	29	35	206
17:00	28	40	206
18:00	27	45	150
19:30	26	50	150
20:00	25	55	65
21:00	24	55	0
22:00	22	60	0
23:00	21	60	0

PPFD: photosynthetic photon flux density
